# Development and Initial Psychometric Evaluation of CIAECSA: A Comprehensive Questionnaire on Attitudes, Experiences, and Contexts of Adolescent Sexuality

**DOI:** 10.3390/healthcare14162544

**Published:** 2026-08-14

**Authors:** Cristina del Rocío Rodríguez-López, Abel Checa-Peñalver, Mónica Raquel Pereira-Afonso, Victoria Lopezosa-Villajos, Isabel Donoso-Calero, Elena Arroyo-Bello, Sagrario Gómez-Cantarino

**Affiliations:** 1 Faculty of Physiotherapy and Nursing of Toledo, University of Castilla-La Mancha, 45004 Toledo, Spain; cristinarocio.rodriguez@alu.uclm.es (C.d.R.R.-L.); abel.checa@uclm.es (A.C.-P.); victoria.lopezosa@alu.uclm.es (V.L.-V.); sagrario.gomez@uclm.es (S.G.-C.); 2 Aula-CuidArte, Faculty of Physiotherapy and Nursing of Toledo, University of Castilla-La Mancha, 45004 Toledo, Spain; mariaisabel.donoso@uclm.es; 3 ENDOCU Research Group, Faculty of Physiotherapy and Nursing of Toledo, University of Castilla-La Mancha, 45004 Toledo, Spain; 4 Faculty of Health Sciences, University of Castilla-La Mancha, Talavera de la Reina, 45004 Toledo, Spain; 5 Fundación Jiménez Díaz School of Nursing, Autonomous University of Madrid (UAM), 28049 Madrid, Spain; elena.arroyo@quironsalud.es

**Keywords:** sexual health, sexuality beliefs, adolescents, questionnaire development, content validity, internal consistency, nursing assessment, subjective perception, self-care

## Abstract

**Highlights:**

**What are the main findings?**

- The CIAECSA questionnaire was developed as an 18-item instrument structured into three theoretically grounded domains related to adolescent sexuality: sexuality/gender/anatomy, emotional/physical pain, and self-care/environment.
- The instrument showed strong content validity, high semantic understandability among adolescents, adequate feasibility, acceptable internal consistency, and an exploratory structure consistent with its theoretical domains.

**What are the implications of the main findings?**

- The CIAECSA may support nurses, educators, and healthcare professionals in identifying misconceptions, educational needs, and psychosocial factors related to adolescent sexual and reproductive health.
- The questionnaire provides a brief and feasible assessment tool that may help guide health education, preventive interventions, and adolescent-centered sexual health promotion in school and community settings.

**Abstract:**

**Background/Objectives:** Adolescent sexuality is multidimensional, yet brief instruments assessing attitudes, experiences, and contextual influences from a biopsychosocial perspective remain limited. This study aimed to develop the Comprehensive Questionnaire on Attitudes, Experiences, and Contexts of Adolescent Sexuality (CIAECSA) and examine its content validity, comprehensibility, feasibility, internal consistency, and exploratory internal structure. **Methods:** A cross-sectional methodological study comprised three phases: theoretical delimitation and generation of 30 initial items; content evaluation by nine experts and semantic validation with 26 adolescents; and pilot administration to 51 adolescents from educational settings in Toledo, Spain. The pilot sample included 35 females (68.6%), 15 males (29.4%), and one gender-fluid participant (2.0%), aged 12–17 years (mean = 14.41; SD = 1.72). Content validity, perceived clarity, feasibility, Cronbach’s alpha, and principal component analysis with Varimax rotation were examined. **Results:** The final questionnaire comprised 18 items across three domains: sexuality/gender/anatomy, emotional/physical pain, and self-care/environment. The scale-level Content Validity Index (S-CVI/Ave) was 0.955, and mean perceived clarity was 4.53/5. The mean completion time was 5.52 min. Cronbach’s alpha was 0.800, 0.780, and 0.740 for the three domains and 0.715 for the total scale. The KMO was 0.659, Bartlett’s test was significant (*p* < 0.001), and an exploratory three-component solution, examined against the prespecified domains, explained 51.4% of the variance. **Conclusions:** The findings provide favorable evidence of content validity, comprehensibility, feasibility, and internal consistency. The CIAECSA may help identify educational needs and psychosocial factors related to adolescent sexuality, although its internal structure and additional measurement properties require examination in larger, balanced, and independent samples.

## 1. Introduction

Sexuality is recognised by the World Health Organisation (WHO) as a fundamental and constitutive dimension of the human being that manifests itself throughout the entire life cycle [1]. Far from being reduced exclusively to reproductive behaviour or the act of intercourse, sexuality complexly integrates biological sex, gender identities and roles, sexual orientation, eroticism, pleasure, intimacy, and affectivity [1,2]. During adolescence, sexuality takes on critical importance, as this is a period of vulnerability and opportunity in which neurobiological maturation coincides with the development of social identity and the awakening of intimate relationships [2].

Recent epidemiological data reinforce the relevance of addressing adolescent sexual health from a multidimensional perspective. In the 2021/2022 Health Behaviour in School-aged Children (HBSC) survey, conducted across 42 countries and regions, 61% of sexually active 15-year-old boys and 57% of girls reported condom use at their last sexual intercourse, whereas 30% of boys and 31% of girls reported that neither a condom nor the contraceptive pill had been used [3]. In Spain, the HBSC-2022 study similarly showed that condom use at the last sexual intercourse decreased from 73.4% among boys and 69.5% among girls aged 15–16 years to 62.9% and 59.9%, respectively, among those aged 17–18 years [4]. These findings illustrate the persistence of gaps in protection and gender- and context-related differences, supporting assessment approaches that extend beyond factual knowledge alone.

The development of the CIAECSA was supported by an integrative conceptual framework rather than by a single explanatory theory. This framework combined the WHO’s holistic definition of sexual health and a biopsychosocial perspective with UNESCO’s comprehensive sexuality education framework and Henderson’s nursing model of basic needs [1,5,6,7]. The WHO and UNESCO frameworks conceptualize sexuality as involving cognitive, emotional, physical, relational, and social dimensions [1,7]. Henderson’s model complements this perspective by emphasizing communication, the expression of emotions and values, learning, self-care, and the avoidance of environmental hazards, all of which are relevant to holistic nursing assessment [5,6]. Together, these perspectives support an assessment that extends beyond biological knowledge and disease prevention to include affective experiences, interpersonal relationships, self-care, and contextual influences.

Within this framework, the CIAECSA operationalizes four closely related but conceptually distinguishable components. Attitudes refer to evaluative positions toward sexuality, affectivity, diversity, and self-care, whereas beliefs refer to cognitive interpretations concerning identity, anatomy, risk, and protective behaviors. Experiences comprise self-reported emotional, physical, and relational responses associated with sexuality, while contexts refer to the perceived influence of family, partners, peers, social networks, and environmental safety [1,2,7]. The questionnaire therefore assesses a multidimensional combination of attitudes, beliefs, perceived experiences, and contextual influences.

Healthy sexual development in youth does not depend solely on the absence of pathologies but on the acquisition of competencies that allow for the establishment of relationships based on respect and autonomy [8,9]. However, this development is subject to the level of knowledge and competencies in sexual education possessed by the individual. The importance of adequate sexual development lies in its direct impact on public health. While an integral sexual sphere fosters self-esteem, poor management of it can lead to risks such as sexually transmitted infections (STIs), unplanned pregnancies, and a distortion of body image [7,10,11,12].

Biological knowledge alone is insufficient without affective education, as affection and emotional bonding are also central to the experience of sexuality [7,13,14,15]. Contemporary assessment also requires the inclusive recognition of diverse gender identities, sexual orientations, and variations in sex characteristics [16,17]. Moreover, the inclusion of emotional distress and pain-related bodily responses is supported by evidence linking adverse peer and romantic relationships to social anxiety and depressive symptoms, while social rejection may share some neural mechanisms with physical pain [18,19]. These findings support considering emotional and bodily responses as interconnected components of adolescent sexual health while avoiding their interpretation as equivalent clinical conditions.

Open communication and trust in reference figures are important protective factors [12,20]. Sexual behavior is also influenced by self-care, resistance to peer pressure, responsible social media use, and substance use [10,21]. Sexual assertiveness supports the communication of needs, consent negotiation, the avoidance of coercive dynamics, and resistance to external expectations, thereby promoting self-care and personal satisfaction [7,21,22]. Existing measures generally focus on specific components of sexual health, such as knowledge and practices among university students [23], sexual self-concept in early adolescent girls [24], or sexual and reproductive empowerment among individuals aged 15–24 years [25]. In contrast, the CIAECSA integrates cognitive-attitudinal foundations concerning sexuality, diversity, and perceived anatomical understanding (Block I) [1,7,16,17]; affective-experiential responses involving communication, rejection, self-esteem, and pain (Block II) [18,19,20]; and behavioral-contextual influences related to self-care, safety, social media, substance use, and peer pressure (Block III) [10,20,21,22]. These complementary domains translate the integrative framework into a brief multidimensional profile for adolescents aged 12–17 years and distinguish the CIAECSA from knowledge tests and single-construct measures, without claiming to exhaustively represent every aspect of adolescent sexuality. Therefore, this study aimed to develop the CIAECSA and conduct an initial psychometric evaluation of its content validity, comprehensibility, feasibility, internal consistency, and exploratory internal structure.

## 2. Materials and Methods

### 2.1. Study Design

A quantitative, observational, and cross-sectional methodological study was conducted to develop and perform an initial psychometric evaluation of the CIAECSA, a questionnaire designed for adolescents aged 12–17 years. The study examined content validity, semantic comprehensibility, feasibility, internal consistency, and the exploratory internal structure of the instrument. The methodological process followed international recommendations for the early stages of health measurement instrument development [26,27,28].

### 2.2. Participants

Three groups participated in the different stages of the study: a multidisciplinary panel of nine professional experts, a semantic-validation sample of 26 adolescents, and a pilot sample of 51 adolescents.

#### 2.2.1. Expert Panel

The expert panel comprised nine professional judges selected according to predefined criteria [29]. Eligible experts were required to have advanced academic training in psychology, nursing, education, sexology, or health sciences. They also needed documented teaching, clinical, or research experience in sexuality-related assessments and previous experience in developing health assessment instruments. Professionals whose experience in these areas could not be documented were excluded.

The panel included a social educator specializing in interventions with minors and gender issues; a nurse holding a PhD and working in pediatric and neonatal care; and a family physician and educator with 22 years of experience. It also included a primary care nurse specializing in community health; a midwife and nurse holding a PhD with 30 years of experience in sexual health; and a social services psychologist specializing in adolescence. The remaining members were a primary education teacher; a health psychologist and neuropsychologist with experience in child and adolescent care; and a secondary school counselor specializing in affective-sexual education programs. This composition provided perspectives from healthcare, education, psychology, and social services.

In addition, a fourth-year nursing student with practical training in community health provided complementary qualitative feedback on item clarity and comprehensibility. This contribution was not included in the CVI calculations, which were based exclusively on the ratings of the nine professional experts.

#### 2.2.2. Semantic Validation Sample

Semantic validation was conducted with a separate convenience sample of 26 adolescents aged 12–17 years, comprising 17 females (65.4%) and 9 males (34.6%). Participants were recruited from the formal educational environment in which the questionnaire was intended to be used. This phase was independent of the subsequent pilot study and was designed to examine item intelligibility and the suitability of the linguistic register for the target population.

#### 2.2.3. Pilot Sample

The pilot evaluation was conducted with a non-probability convenience sample of 51 adolescents recruited from educational settings in the province of Toledo, Castilla-La Mancha, Spain. Participants were enrolled in secondary education or the first year of upper-secondary education (Bachillerato). They were aged 12–17 years, with a mean age of 14.41 years (SD = 1.72). The sample included 35 females (68.6%), 15 males (29.4%), and one participant who identified as gender-fluid (2.0%). The sample was not designed to be representative of the general adolescent population, and its sex/gender distribution was asymmetric.

The pilot sample size was established according to the operational objectives of this phase. Hertzog’s recommendations indicate that samples of approximately 30–40 participants may be sufficient for evaluating feasibility, response time, and initial item performance [30]. According to Viechtbauer’s formula, a sample of 51 participants provides an estimated 92.7% probability of detecting at least one problem occurring in 5% of the target population [31]. This rationale applies to the feasibility and problem-detection objectives of the pilot study and not to definitive evaluation of the questionnaire’s dimensional structure (Table 1).

#### 2.2.4. Eligibility Criteria

The inclusion criteria for the adolescent phases were: (1) being aged 12–17 years at the time of participation; (2) having sufficient command of Spanish to understand and answer the questionnaire; (3) having informed consent provided by a parent or legal guardian; and (4) providing assent to participate. Exclusion criteria were: (1) being outside the predefined age range; (2) absence or withdrawal of parental consent or adolescent assent; (3) cognitive, linguistic, sensory, or comprehension-related difficulties that prevented autonomous and reliable completion of the questionnaire; and (4) returning the questionnaire without responses.

### 2.3. Instrument Development and Scoring

The development process began with a four-month review of the scientific literature to delimit the theoretical construct and identify relevant domains and indicators. The review included PubMed/MEDLINE, Scopus, Web of Science, CINAHL, PsycINFO, and ERIC and examined evidence concerning adolescent sexual development, associated biopsychosocial factors, and affective-sexual education.

Based on this conceptual foundation, an initial pool of 30 items was generated and organized into three theoretically defined domains. Block I, sexuality, gender, and anatomy, addressed conceptions of sexuality and affectivity, respect for diversity, gender identity, perceived anatomical understanding, and variations in sex characteristics. Block II, sexuality and emotional/physical pain, addressed affective rejection, emotional distress, bodily responses, communication with reference figures, self-esteem, and pain. Block III, sexuality and self-care/environment, addressed assertiveness, environmental safety, partner communication, substance-related risk, social media, and peer pressure.

Item development followed an inclusive and non-pathologizing approach intended to represent different identities and orientations. A five-point Likert-type response format was selected, ranging from 1 (strongly disagree) to 5 (strongly agree), to provide sufficient response differentiation while maintaining a manageable format for adolescents [32,33,34].

Following expert evaluation, 12 items were removed because of insufficient agreement, redundancy, limited construct specificity, developmental inappropriateness, excessive technical complexity, contextual inadequacy, or limited applicability to the complete target population. The refinement process resulted in a final version of 18 items, with six items assigned to each theoretical domain. Items were retained according to their conceptual representativeness, clarity, pertinence, relevance, non-redundancy, developmental suitability, and contribution to the coverage of the three domains (Table 2).

Items I-3 and I-6 were retained after qualitative review because the expert panel considered them conceptually pertinent and relevant despite their lower clarity ratings. Both items were identified as requiring further age-appropriate cognitive testing. The domain assignment, content facet, and wording of the final 18 items are presented in Table 3.

Each item is rated from 1 to 5. Items 1.3, 2.1, and 3.6 are reverse-coded. After recoding, the six items within each domain can be summed to obtain domain-specific scores ranging from 6 to 30. The 18 items can also be summed to obtain a descriptive total score ranging from 18 to 90. Higher scores indicate greater alignment with the conceptual direction of the questionnaire. No diagnostic cut-off values were evaluated in the present study.

### 2.4. Procedure

#### 2.4.1. Ethical Approval, Recruitment, and Administration

The study was approved by the Social Research Ethics Committee of the University of Castilla-La Mancha (CEIS-661803-V4Z4). Participating secondary schools were contacted by the research team. After authorization was obtained from the administration of each school, information about the study was provided to teaching staff, families, and students. Informed consent was obtained from parents or legal guardians, and assent was obtained from the adolescents before participation. Recruitment was conducted through participating classrooms, and all students within the predefined age range were invited to participate. Data were collected in person using a paper-based questionnaire administered under the supervision of members of the research team.

#### 2.4.2. Phase 1: Conceptual Delimitation and Item Generation

During the first phase, the theoretical construct was delimited through the literature review described in Section 2.3. Relevant indicators were extracted and used to generate the initial 30-item pool, which was organized according to the three prespecified domains.

#### 2.4.3. Phase 2: Expert Evaluation and Semantic Validation

A formal electronic invitation was sent to the nine professional experts together with the validation protocol, the theoretical foundation of the questionnaire, and the evaluation form. Experts were given 30 days to complete their assessments independently [29]. Each of the 30 initial items was evaluated using a five-point ordinal scale and three criteria. Clarity referred to semantic comprehensibility and the absence of ambiguity. Pertinence assessed the suitability of the item for its assigned domain, whereas relevance reflected its importance and utility for the target population and the clinical-educational context.

Quantitative ratings and qualitative observations were subsequently reviewed to identify items requiring elimination, reformulation, or retention. This process resulted in the removal of 12 items and the consolidation of the 18-item version.

The 18-item version was subsequently administered to a separate sample of 26 adolescents. Participants evaluated the perceived clarity of the items using a five-point scale and provided qualitative observations concerning wording and interpretation. This phase was intended to identify semantic or grammatical difficulties before pilot administration.

#### 2.4.4. Phase 3: Pilot Administration

The final 18-item version was administered to the pilot sample of 51 adolescents. Administration was conducted in paper format, in person, and under the supervision of members of the research team. The pilot assessment was completed at baseline, before the implementation of any educational intervention. Participants completed the CIAECSA and were provided with open-ended observation fields after each block. Completion time, acceptability, response behavior, internal consistency, and exploratory internal structure were examined.

### 2.5. Statistical Analysis

Data were processed using IBM SPSS Statistics version 24 and Microsoft Excel. Quantitative variables were summarized using the mean, standard deviation, and observed range, whereas categorical variables were summarized using absolute and relative frequencies.

#### 2.5.1. Content Validity

Content validity was examined using the Content Validity Index (CVI) [27,35]. Expert ratings of 4 or 5 were classified as favorable. The Item-level Content Validity Index (I-CVI), average Domain-level Content Validity Index (D-CVI/Ave), and average Scale-level Content Validity Index (S-CVI/Ave) were calculated from the ratings of the nine professional experts. An I-CVI threshold of at least 0.78 was established, corresponding to at least seven favorable ratings in a nine-member panel [27,35]. Modified kappa coefficients were also calculated to estimate chance-corrected agreement for each item and criterion [35].

#### 2.5.2. Semantic Comprehensibility and Feasibility

During semantic validation, ratings of 4 or 5 on the five-point perceived-clarity scale were classified as favorable. Item-level comprehensibility was calculated as the proportion of participants providing a favorable rating, and a minimum agreement threshold of 80% was established. Mean clarity scores were also calculated for each domain and for the questionnaire overall. Participants’ qualitative observations were reviewed descriptively to identify grammatical or interpretative difficulties. Pilot feasibility was examined through completion time, acceptability, completeness of item responses, and the qualitative observations provided after each domain.

#### 2.5.3. Statistical Assessment of Internal Consistency and Component Structure

Internal consistency was estimated using Cronbach’s alpha for each theoretical domain and for the complete 18-item questionnaire after reverse coding items 1.3, 2.1, and 3.6. Item–total correlations and alpha-if-item-deleted values were inspected to identify items requiring further monitoring. Cronbach’s alpha was retained as a conventional initial estimate that facilitates comparison with previous early-stage instrument-development studies [28,36]. Because CIAECSA uses five-point ordinal response categories, the coefficients were interpreted cautiously. Complementary coefficients suitable for ordinal data, including McDonald’s omega and ordinal alpha based on polychoric correlations, should be examined in larger samples.

The adequacy of the pilot data for exploratory inspection was assessed using the Kaiser–Meyer–Olkin index and Bartlett’s test of sphericity. Principal component analysis (PCA) with Varimax rotation was performed, and component loadings above 0.40 were considered salient. A three-component solution was examined because the questionnaire had been developed according to three prespecified theoretical domains.

PCA was selected as an exploratory and descriptive procedure to inspect how the total variance of the item responses was organized and whether the observed component pattern broadly corresponded to the prespecified domains. It was not intended to estimate or confirm a latent factor model. Because the pilot sample size was established for feasibility and problem-detection purposes, the component solution was interpreted cautiously and not as definitive evidence of structural validity. Common-factor exploratory factor analysis based on polychoric correlations, followed by confirmatory factor analysis in a larger independent sample, will be required to evaluate the latent structure of the CIAECSA. Test–retest reliability, convergent, discriminant, and criterion validity, responsiveness, and measurement invariance were not evaluated in this phase.

## 3. Results

### 3.1. Results of Content and Face Validation with the Evaluation Panel

The final expert panel consisted of nine professional judges with extensive clinical, teaching, and research experience in the fields of sexuality, health, and education. The CVI analyses were calculated exclusively from the ratings provided by these nine experts. In addition, a fourth-year nursing student with training in community health provided complementary qualitative feedback on item clarity and understandability, offering a generational perspective closer to the target population. This complementary contribution was used to inform face-validity considerations but was not included in the quantitative CVI computation.

Content validity was evaluated using the Item-level Content Validity Index (I-CVI). For the analysis, the categories “High” and “Very high” (4 and 5) were considered favorable responses. The I-CVI values ranged between 0.56 and 1.00, with an overall Scale-level CVI average (S-CVI/Ave) of 0.955, providing initial support for content validity. The S-CVI/Ave was 0.926 for clarity, 0.969 for pertinence, and 0.969 for relevance. Following the recommendations for a panel of nine judges, an I-CVI ≥ 0.78 (equivalent to a minimum of 7 favorable agreements) was adopted as the acceptability criterion [27,35].

Under this criterion, item I-3 in the clarity dimension (I-CVI = 0.56) and item I-6 in the clarity dimension (I-CVI = 0.67) were identified as the main elements requiring qualitative review and specific monitoring during the pre-test application (Table 4). In addition, item I-6 showed borderline values for pertinence and relevance (I-CVI = 0.78 in both criteria), supporting the need for specific qualitative justification rather than automatic deletion.

Regarding the clarity criterion, most items achieved optimal Content Validity Indices (CVI), ranging between 88.9% and 100.0%. This performance suggests that the terminology used possesses an adequate level of intelligibility for the target population. However, items I-3 (55.6%) and I-6 (66.7%) were found to have scores lower than the 0.78 CVI threshold. Consequently, these items underwent technical re-evaluation and qualitative review aimed at optimizing the interpretation of the findings and mitigating the ambiguities identified by the evaluation committee.

In terms of pertinence, the panel’s assessment supported the suitability of the items for measuring the underlying theoretical constructs, with CVI values of 100.0% recorded in virtually all evaluated blocks. Only item I-6 (77.8%) was at the lower limit of the established cutoff point, suggesting, in general, an adequate correspondence between the indicators and the conceptual dimensions of the instrument.

Similarly, the relevance criterion exhibited high validity levels, mostly ranging between 88.9% and 100.0%. The only exception was again item I-6, which reached a value of 77.8%. These findings corroborate that the instrument’s content is substantive and representative for both the clinical context and the study population.

Finally, those items that did not exceed or were strictly at the limit of the acceptance criterion (I-CVI ≥ 0.78) were subject to additional qualitative analysis. The review derived from this process, based on the panel’s consensus, focused on the methodological function of inverse wording and on the clarification of technical terminology. The mean item-level CVI across the three criteria ranged between 74.1% and 100.0% (Table 5).

The removal of any final questionnaire items was not necessary, as the panel considered all items to be conceptually pertinent and relevant. Following the discussion and consensus phase, in which qualitative observations were analyzed, the final version of the CIAECSA questionnaire was consolidated. This version maintains the integrity of its original structure and the total number of items (*n* = 18), ensuring that all dimensions of the construct are duly represented.

Overall, the results provide support at this methodological stage for the suitability of the CIAECSA questionnaire in terms of clarity, pertinence, and relevance. The global S-CVI/Ave (95.5%) and the observed level of agreement support the instrument’s content validity at this developmental stage. At the dimension level, the D-CVI/Ave values were 90.1% for Block I, 98.8% for Block II, and 97.5% for Block III. Modified kappa coefficients ranged from 0.41 to 1.00, indicating fair-to-excellent chance-corrected agreement. Items I-3 and I-6 were retained because of their theoretical relevance. However, their lower clarity scores indicate the need for further age-appropriate cognitive testing.

In conclusion, the results obtained provide favorable methodological support for the development of the CIAECSA, especially concerning its content validity, semantic understandability, and feasibility of application. Further studies should examine its temporal stability, construct validity, and sensitivity to change in larger and more diverse adolescent samples.

### 3.2. Results of Semantic Validation

Following validation by the expert panel, the CIAECSA instrument was subjected to a process of semantic and face validation involving a convenience sample of 26 young people (*n* = 26) from the formal educational environment in which its application is intended. This phase, qualitative and quantitative in nature, aimed primarily to assess the intelligibility of the items and to ensure the linguistic register was suitable for the target participants, constituting a complementary step independent of the pilot study. The findings provide evidence of comprehensibility in the target age group, although they should not be interpreted as proof of representativeness for the entire adolescent population.

In the quantitative comprehension analysis, an overall mean score of 4.53 out of 5 was obtained on the perceived clarity scale. Scores were also high across the three domains. Mean values were 4.56 for Block I (sexuality, gender, and anatomy), 4.40 for Block II (sexuality and emotional/physical pain), and 4.63 for Block III (sexuality and self-care/environment). These values indicate a high overall level of perceived comprehensibility across the three questionnaire blocks.

Complementarily, the qualitative analysis of observations provided by participants allowed for minor grammatical adjustments to optimize the instrument’s flow. Overall, the high comprehension mean and the qualitative refinement performed provide initial support for the face validity of CIAECSA and suggest that the instrument is technically applicable in adolescent educational and health-promotion contexts.

### 3.3. Results of the Pilot Test

To evaluate feasibility, semantic clarity, and the measurement behavior of the instrument, a pilot study was conducted with a sample of 51 adolescents (*n* = 51). This phase was limited to baseline measurement prior to the implementation of any training or educational intervention. The analysis provides evidence on acceptability, response behavior, internal consistency, and exploratory structure in the studied sample.

The pilot sample included 35 adolescent females (68.6%), 15 adolescent males (29.4%), and one person identifying as gender-fluid (2.0%). Ages ranged from 12 to 17 years, with a mean of 14.41 years (SD = 1.72). The age distribution was approximately balanced across the 12–17-year range, although the sex/gender distribution was asymmetric, with a higher proportion of female participants. The age distribution was as follows: 12 years (*n* = 9; 6 females, 3 males), 13 years (*n* = 9; 6 females, 3 males), 14 years (*n* = 9; 6 females, 3 males), 15 years (*n* = 8; 6 females, 2 males), 16 years (*n* = 8; 5 females, 2 males, 1 gender-fluid), and 17 years (*n* = 8; 6 females, 2 males).

The sample size was determined following Hertzog’s criteria, which suggest that for instrument development studies, a size of 30 to 40 participants is sufficient to assess feasibility, response time, and initial item stability [30]. Likewise, according to Viechtbauer’s formula, a sample of 51 participants provides approximately a 92.7% probability of detecting at least one comprehension problem or ambiguity affecting 5% of the evaluated population; a sample close to 59 participants would be required to reach approximately 95% probability. Therefore, the current sample is considered sufficient for the operational objectives of the pre-test, although it does not imply population representativeness nor allow for the generalization of results across the entire target age range [31].

The results indicated that the questionnaire was well received in this pre-training context focused on sex education. The estimated completion time averaged 5.52 min, and 38 participants (74.5%) completed the questionnaire in approximately 4 to 5 min, suggesting that the 18-item length is generally appropriate for maintaining attention and reducing fatigue in this adolescent sample.

With regard to face validity, the open-ended observation fields provided after each block did not reveal substantial spontaneous reports of ambiguity, supporting the general appropriateness of the language within the studied sample. Internal consistency was assessed in this revision as an exploratory measurement indicator because the database provided sufficient complete responses for all 18 items.

These results support the usefulness of the questionnaire as a development tool for obtaining a general baseline profile of the subgroup studied. They also justify moving forward with additional measurement studies using more diverse and balanced samples, including test–retest reliability, convergent validity, and confirmatory structural analysis.

### 3.4. Internal Consistency and Exploratory Component Analysis

After reverse coding items 1.3, 2.1, and 3.6, the internal consistency coefficients were acceptable for all three theoretical blocks and for the full 18-item scale. Cronbach’s alpha was 0.800 for Block I, 0.780 for Block II, 0.740 for Block III, and 0.715 for the total scale (Table 6). The descriptive total scores suggested higher mean scores in Block I and Block III than in Block II, indicating that emotional/physical pain may represent the domain requiring greater educational attention in future interventions.

Because the pilot sample comprised 51 participants, the PCA was treated as a descriptive exploratory inspection rather than as a definitive evaluation of dimensionality. The KMO index was 0.659, and Bartlett’s test of sphericity was statistically significant (χ^2^(153) = 309.67; *p* < 0.001). These indicators supported the presence of sufficient inter-item correlations to conduct an exploratory inspection; however, they do not overcome the limitations imposed by the sample size. Although five components had eigenvalues greater than 1, a three-component solution was examined because the questionnaire had been developed according to three prespecified theoretical domains. This solution explained 51.4% of the total variance and should be considered provisional and sample-dependent.

The rotated solution broadly reproduced the theoretical blocks. Items 1.1 to 1.6 loaded primarily on the first component; items 2.1 to 2.6 loaded primarily on the second component; and items 3.2 to 3.6 loaded primarily on the third component. Item 3.1 showed a cross-loading pattern, which may reflect its broader conceptual nature as a general self-care statement.

## 4. Discussion

The findings support the content coverage, comprehensibility, and feasibility of the CIAECSA while also identifying specific areas requiring further methodological refinement. Taken together, the results provide a coherent basis for continued evaluation of the questionnaire. Further studies with larger and independent samples are required to confirm its structural validity and extend the evidence regarding additional measurement properties.

Content validity was supported by the high overall S-CVI/Ave of 0.955 and by the fact that most I-CVI values met or exceeded the recommended threshold of 0.78 for a panel of nine experts [27,35,37]. However, two items from Block I warrant further cognitive exploration. Item I-3 may combine two related propositions—attraction to different genders and equal respect—and does not clarify whether attraction is understood as simultaneous or as occurring at different times. Item I-6 may depend on respondents’ familiarity with the term “intersex”, particularly among younger adolescents, while “natural” may be interpreted as “common” or “frequent”. The inclusion of I-6 was intended to ensure conceptual coverage of variations in sex characteristics rather than to mirror population prevalence; nevertheless, its relative contribution to Block I should be examined in larger samples. Although the semantic-validation findings showed high overall comprehensibility, these item-specific issues should be examined in subsequent questionnaire-evaluation studies.

Future studies should conduct age-appropriate cognitive interviews with adolescents across the 12–17-year range. Respondents should be asked which questions make the least sense or appear confusing and why and should be invited to paraphrase the items and explain how they selected their responses.

Comparison with existing instruments helps clarify the specific contribution of the CIAECSA. The questionnaire developed by León-Larios and Gómez-Baya primarily assesses sexual and contraceptive knowledge and practices among university students [23], whereas the Sexual Self-Concept Inventory focuses on sexual arousability, sexual agency, and negative sexual affect in early adolescent girls [24]. The Sexual and Reproductive Empowerment Scale for Adolescents and Young Adults shares CIAECSA’s multidimensional orientation and addresses related areas such as partner communication, parental support, sexual safety, and self-love, but it was developed for individuals aged 15–24 years and comprises seven specific subscales [25]. In contrast, the CIAECSA integrates self-concept, communication, diversity, emotional and bodily responses, self-care, digital influences, substance-related risk, and peer pressure in a brief questionnaire for adolescents aged 12–17 years. Differences in the constructs assessed, target populations, and stages of questionnaire development make direct comparisons between their reliability coefficients inappropriate.

The domain-level Cronbach’s alpha coefficients indicate internal coherence at this stage but do not establish the dimensionality of the questionnaire. The lower coefficient for the total score may reflect heterogeneity across the three domains and supports prioritizing domain-specific interpretation, although this pattern alone cannot demonstrate multidimensionality. The PCA showed broad correspondence with the prespecified domains; however, the presence of five components with eigenvalues greater than 1 and the cross-loading of item 3.1 indicate that alternative structural solutions remain plausible. Therefore, the three-component pattern should guide subsequent factor-analytic evaluation rather than be interpreted as confirmation of the CIAECSA’s dimensional structure.

At the present stage of development, domain-specific scores should constitute the primary level of interpretation. After reverse coding items 1.3, 2.1, and 3.6, each domain produces a score ranging from 6 to 30 and can be used descriptively to identify patterns relating to cognitive-attitudinal foundations, affective-experiential responses, and behavioral-contextual influences. The total score, ranging from 18 to 90, may also be reported for descriptive purposes, but it should not currently be interpreted as evidence that the CIAECSA represents a unidimensional construct. Neither domain-specific nor total scores should be used diagnostically, and no clinical or educational cut-off values can yet be recommended. Further structural and validity studies should determine whether interpretation of a global score is psychometrically supported.

From a theoretical perspective, the findings support further examination of adolescent sexuality through complementary cognitive-attitudinal, affective-experiential, and behavioral-contextual domains. This organization is consistent with the biopsychosocial and comprehensive sexual health frameworks underlying the CIAECSA, although its empirical structure requires confirmation in larger samples. From a practical perspective, the questionnaire may support the structured identification of educational needs [38,39,40] relating to diversity, communication [41], emotional distress [42], self-care, digital influences, and peer pressure. At its current stage, however, it should be considered an educational and research resource rather than a diagnostic instrument or a tool for classifying individual adolescents. Its results should be interpreted alongside the adolescent’s developmental, family, educational, and sociocultural context [43,44].

Several limitations restrict the interpretation and generalizability of the present findings. First, although the pilot sample was appropriate for examining feasibility, comprehensibility, response behavior, and initial problem detection, a sample of 51 adolescents does not provide a sufficiently robust basis for stable estimation of dimensionality. The limited sample may also reduce the precision of the internal consistency coefficients and produce sample-dependent component loadings. The KMO index and Bartlett’s test indicate the presence of inter-item correlations but do not compensate for this sample-size limitation. Second, convenience sampling from educational settings in a single Spanish province limits the representativeness of the sample and may have introduced selection bias. Third, the predominance of female participants may have influenced item distributions, inter-item correlations, reliability estimates, and the observed component pattern, particularly for content related to gender, relationships, safety, and self-care. The small numbers of male and gender-diverse participants prevented meaningful subgroup comparisons and measurement invariance analyses.

Fourth, the same pilot sample was used for the exploratory component analysis, and no independent sample was available to examine the reproducibility of the proposed structure. Fifth, temporal stability, convergent validity, discriminant validity, and criterion validity were not assessed. Responsiveness to educational interventions or change over time was also not examined. Finally, internal consistency was estimated using Cronbach’s alpha; complementary coefficients appropriate for ordinal data, including McDonald’s omega and ordinal alpha calculated from polychoric correlations, should be examined in subsequent studies.

Subsequent studies should therefore recruit larger, more heterogeneous samples with a better balance across sex/gender groups; conduct common-factor EFA based on polychoric correlations followed by CFA in an independent sample; estimate complementary reliability coefficients; and examine test–retest reliability, convergent, discriminant, and criterion validity, responsiveness, and measurement invariance across relevant age and sex/gender groups.

## 5. Conclusions

This study developed the CIAECSA, a brief, theoretically grounded questionnaire designed to assess attitudes, experiences, and contextual influences related to sexuality among adolescents aged 12–17 years. The combination of multidisciplinary expert judgment, semantic evaluation with adolescents, and pilot administration provides favorable evidence of content validity, comprehensibility, feasibility, and internal coherence within the three proposed domains. Its concise, multidimensional, and age-specific design supports its potential value as an educational and research resource for informing the structured assessment of adolescent sexual health needs.

The findings should nevertheless be interpreted within the methodological scope of this study. The exploratory component analysis and the limited convenience sample do not confirm the dimensional structure of the CIAECSA. At its current stage, domain-specific scores should constitute the primary level of interpretation, while the total score may be reported descriptively. Larger, more heterogeneous, sex/gender-balanced, and independent samples are required to examine the questionnaire’s factor structure, temporal stability, construct validity, responsiveness, and measurement invariance.

## Figures and Tables

**Table 1:** Characteristics of the adolescent samples involved in semantic validation and pilot testing.

Characteristic	Semantic Validation	Pilot Testing
Sample size	26	51
Age range	12–17 years	12–17 years
Age, mean (SD)	14.77 (1.68)	14.41 (1.72)
Female	17 (65.4%)	35 (68.6%)
Male	9 (34.6%)	15 (29.4%)
Gender-fluid	0	1 (2.0%)
Geographical setting	Toledo, Castilla-La Mancha, Spain	Toledo, Castilla-La Mancha, Spain

Note: Data are presented as *n* (%) unless otherwise indicated. SD = standard deviation.

**Table 2:** Items excluded after expert judgment and reasons for exclusion from the initial item pool.

Domain (Block)	Item	Reason for Exclusion
I. Sexuality, gender and anatomy.	I know the names of Sexually Transmitted Infections.	Assessment of rote knowledge.
	I believe that men should always take the first step in a relationship.	Heteronormativity and binarism bias.
	I would like to have children when I am an adult.	Lack of temporal relevance.
	Puberty is the most difficult stage of life.	Lack of thematic specificity.
II. Sexuality and emotional/physical pain.	I feel sad if I do not have a steady partner at this moment.	Lack of construct specificity.
	Sometimes I feel jealous if I see my partner talking to other people.	Theoretical inadequacy.
	I think about sex many times throughout the day.	Invasiveness and low relevance.
	I usually cry when I have affective or relationship problems.	Low discriminatory power.
III. Sexuality and self-care/environment.	I avoid partying with people I don’t know to avoid sexual risks.	Construct confusion.
	I know the legal steps to take in the event of an assault.	Excessive technical complexity.
	I usually read news about sexual health in newspapers or magazines.	Contextual inadequacy and anachronism.
	I always carry a condom with me in case a relationship arises.	Bias due to sexual debut and lack of universal applicability.

Note: Item-removal decisions were based on multidisciplinary expert review and considered construct specificity, developmental appropriateness, contextual relevance, gender neutrality, and applicability across the target population.

**Table 3:** Specification matrix and final CIAECSA items by domain and content facet.

ID	Content Facet	Item
Block I. Sexuality, gender, and anatomy.		
1.1	Concept of Sexuality	My sexuality depends on my emotions, relationships, and tastes, and is not limited only to the sexual act.
1.2	Affectivity	I consider that words of affection or displays of affection, such as holding hands, are part of sexuality.
1.3 *	Diversity and Respect	I believe that people cannot be attracted to different genders or sexual orientations and therefore do not deserve the same respect.
1.4	Gender Identity	I believe there can be a difference between my birth sex and the gender I identify with.
1.5	Anatomical Knowledge	I feel capable of explaining the general function of the genitals.
1.6	Biological Variations	I consider that being born with intersex characteristics is something natural.
Block II. Sexuality and emotional/physical pain.		
2.1 *	Affective Resilience	I do not believe that criticism or judgments about my affective or sexual life cause me emotional pain.
2.2	Somatization	When I feel emotionally rejected, my body reacts with symptoms such as headaches, stomach discomfort, or anxiety.
2.3	Family Communication	The lack of confidence to talk about sexuality with my parents or other adults causes me internal distress.
2.4	Self-concept	Emotions linked to attraction or love have a strong impact on my self-esteem and how I perceive my body.
2.5	Risk Perception	When I think about sexuality, I also think about the risk of diseases and physical discomfort.
2.6	Impact of Pain	Sexual experiences that generate physical pain can affect self-esteem.
Block III. Sexuality and self-care/environment.		
3.1	Assertiveness	Self-care is not only about physical protection, but also about knowing how to say ‘no’.
3.2	Environmental Safety	In my environment, talking about gender identity is safe.
3.3	Risky Behaviors	When people use recreational drugs, they tend to take more risks in their sexual lives.
3.4	Partner Communication	Having clear communication with my partner is part of my sexual self-care.
3.5	Digital Influence	Social networks influence how I understand and experience my sexuality.
3.6 *	Peer Pressure	I believe that peer pressure does not affect my ability to practice self-care in my sexuality.

Note: All items are rated using a five-point Likert-type scale (1 = strongly disagree; 5 = strongly agree). Items 1.3, 2.1, and 3.6 are reverse-coded. * Negatively worded and reverse-scored items included to reduce acquiescence bias.

**Table 4:** Summary of Content Validity Index values by criterion (I-CVI).

Block	Item No.	Clarity (*n*)	I-CVI %	Pertinence (*n*)	I-CVI %	Relevance (*n*)	I-CVI %	Mean I-CVI (%)
I	1	9	100.0%	9	100.0%	9	100.0%	100.0%
I	2	9	100.0%	9	100.0%	8	88.9%	96.3%
I	3	5	55.6%	9	100.0%	9	100.0%	85.2%
I	4	8	88.9%	8	88.9%	8	88.9%	88.9%
I	5	9	100.0%	9	100.0%	8	88.9%	96.3%
I	6	6	66.7%	7	77.8%	7	77.8%	74.1%
II	1	8	88.9%	9	100.0%	9	100.0%	96.3%
II	2	9	100.0%	9	100.0%	9	100.0%	100.0%
II	3	8	88.9%	9	100.0%	9	100.0%	96.3%
II	4	9	100.0%	9	100.0%	9	100.0%	100.0%
II	5	9	100.0%	9	100.0%	9	100.0%	100.0%
II	6	9	100.0%	9	100.0%	9	100.0%	100.0%
III	1	9	100.0%	9	100.0%	9	100.0%	100.0%
III	2	8	88.9%	8	88.9%	9	100.0%	92.6%
III	3	9	100.0%	9	100.0%	9	100.0%	100.0%
III	4	9	100.0%	9	100.0%	9	100.0%	100.0%
III	5	9	100.0%	9	100.0%	9	100.0%	100.0%
III	6	8	88.9%	8	88.9%	9	100.0%	92.6%

Note: *n* = number of experts with a favorable rating (score 4–5); I-CVI = Item-level Content Validity Index. Items with low or borderline I-CVI values were qualitatively reviewed by the panel; no item was removed from the final version at this stage. Modified kappa values were calculated from dichotomized ratings and ranged from 0.41 to 1.00.

**Table 5:** Qualitative review and panel decisions following the panel evaluation (items with low or borderline I-CVI values).

Item	Affected Dimension	Original Wording	Modification Made	Justification
I-3	Clarity	I believe that people cannot be attracted to different genders or orientations and therefore do not deserve the same respect.	Retained; further cognitive testing recommended.	Low clarity (0.56), despite optimal pertinence and relevance (1.00). Retained for theoretical relevance, but its double-barreled wording requires further cognitive testing.
I-6	Clarity, Pertinence, and Relevance	I consider that being born with intersex characteristics is something natural.	Retained; further cognitive testing recommended.	Low clarity (0.67) and borderline pertinence/relevance (0.78). Retained for conceptual relevance; understanding of “intersex” and “natural” requires further cognitive testing.

Note: The dimensional structure and item distribution result from the content validation process through expert judgment and the calculation of the Content Validity Index (CVI).

**Table 6:** Internal consistency indicators and descriptive scores by CIAECSA domain.

Domain	No. of Items	Cronbach’s Alpha	Mean Total Score (SD)	Observed Range
Block I. Sexuality, gender, and anatomy	6	0.800	23.18 (5.17)	9–30
Block II. Sexuality and emotional/physical pain	6	0.780	20.27 (4.98)	11–30
Block III. Sexuality, self-care, and environment	6	0.740	22.92 (4.03)	12–30
Full CIAECSA scale	18	0.715	66.37 (8.56)	41–83

Note: Scores were calculated using the three domains and following the reverse recoding of items 1.3, 2.1, and 3.6. Higher scores indicate greater alignment with the conceptual direction of the instrument.

## Data Availability

The original contributions presented in this study are included in the article. Further inquiries can be directed to the corresponding author.

## Abbreviations

The following abbreviations are used in this manuscript:

CIAECSA	Comprehensive Questionnaire on Attitudes, Experiences and Contexts of Sexuality among Adolescents
CVI	Content Validity Index
I-CVI	Item-level Content Validity Index
S-CVI	Scale-level Content Validity Index
STI	Sexually Transmitted Infections
